# Patterns of ocular trauma in elderly patients in an urban population-the Bronx experience

**DOI:** 10.5935/0004-2749.20200025

**Published:** 2020

**Authors:** Isaac M. Chocron, Lediana Goduni, David M. Poulsen, Joyce N. MbekeanI

**Affiliations:** 1 Department of Ophthalmology, Emory School of Medicine, Atlanta, GA, USA; 2 Department of Ophthalmology, NYU Langone Health, New York, NY, USA; 3 Department of Ophthalmology & Visual Sciences, Montefiore Medical Center/Albert Einstein College of Medicine, Bronx, NY, USA; 4 Department of Surgery (Ophthalmology), Jacobi Medical Center, Bronx, NY, USA

**Keywords:** Eye injuries, Epidemiology, Age, Accidental falls, Urban population, Traumatismos oculares/epidemiologia, Idoso, Acidentes por quedas, População urbana

## Abstract

**Purpose:**

To evaluate the characteristics of ocular injuries among elderly patients admitted to an urban level I trauma center because of major trauma from 2008 to 2015.

**Methods:**

A retrospective chart review was conducted of patients aged >65 years admitted with ocular injuries that were identified with ICD-9 codes. Tabulated data were analyzed using the Student’s paired *t*-test, the chi-squared test, and regression analysis using STATA/MP-12 software. Significance was set at *p*<0.05.

**Results:**

Of a total of 861 patients, 221 (25.7%) admitted for major trauma and ocular injuries were elderly. The mean age of these patients was 80.3 years (median =79.2 years; interquartile range=63.8-94.6 years). Of these patients, 40.7% were males and 59.3% were females. The males were younger than the females (mean age, 77.3 vs. 82.4 years, respectively, p<0.001). Race was documented as white (30.8%), black (13.6%), and “other” (54.3%), with 67.5% of the “other” group (36.7% overall) identified as Hispanic. The most frequent injuries were contusion of the eye/ adnexa (68.2%), orbital wall fractures (22.2%), and an open wound of the ocular adnexa (18.1%). Males had a 2.64-fold greater risk of orbital wall fractures (95% confidence interval [CI]=1.38-5.05, p<0.003). Patients with orbital wall fractures had higher injury severity scores than those without (95% CI=14.1-20.9 vs. 6.8-8.6, respectively, p<0.001). The most common injuries were falls (77.8%) and pedestrian/motor vehicle accidents (6.8%). Falls occurred mostly at home (51.7%), on the street (13.9%), and in hospitals/nursing homes (12.2%). Those falling at home were older than those falling at other locations (95% CI=81.8-85.4 vs. 77.0-80.6 years, respectively, p<0.002).

**Conclusions:**

Ocular injuries in elderly Bronx patients most commonly occurred in females due to falls in the home/nursing home setting. Public health measures addressing identifiable individual and environmental risks in these common locations would be most beneficial in reducing the incidence of ocular injuries in this population.

## Abstract (pt)

**Objetivo:**

Avaliar as características das lesões oculares de idosos nas internações por grandes traumatismos em um centro urbano de trauma nível I de 2008 a 2015.

**Métodos:**

Realizou-se uma revisão retrospectiva de prontuários de pacientes com mais de 65 anos internados com lesões oculares identificados com os códigos CID-9. Os dados tabulados foram analisados com o teste t de Student, teste qui-quadrado e análise de regressão, utilizando o software STATA/MP-12. A significância estatística foi fixada em p<0,05.

**Resultados:**

Duzentos e vinte e um (25,7%) pacientes de um total de 861, admitidos por traumatismo craniano importante e lesões oculares, eram idosos. A idade média era de 80,3 anos (mediana=79,2; intervalo interquartil=63,8-94,6). 40,7% eram do sexo masculino e 59,3% do feminino. Os homens eram menos idosos (média=77,3) do que as mulheres (média=82,4), p<0,001. A raça foi documentada como branca (30,8%), negra (13,6%) e “outra” (54,3%); 67,5% dos “outros” (36,7% no geral) identificados como hispânicos. As lesões mais frequentes foram contusão do olho/anexos (68,2%), fraturas da parede orbital (22,2%) e ferida aberta dos anexos oculares (18,1%). Os homens tiveram 2,64 mais chances de fraturas da parede orbital (95% CI=1,38-5,05; p<0,003). Pacientes com fraturas da parede orbital tiveram maiores escores de gravidade da lesão (95% CI=14,1-20,9) do que aqueles sem fraturas (96% IC=6,8-8,6), p<0,001. Os mecanismos comuns foram quedas (77,8%) e acidentes a pé com veículos automotores (6,8%). As quedas ocorreram principalmente em casa (51,7%), na rua (13,9%) e em hospitais/lares de idosos (12,2%). Aqueles que caíram em casa eram mais velhos (IC 95%=81,8-85,4) do que os que tiveram quedas em outros locais (IC 95%=77,0-80,6), p<0,002.

**Conclusões:**

Lesões oculares em pacientes idosos de Bronx foram mais comuns no sexo feminino e devido a quedas que ocorreram em casa/lar de idosos. Medidas de saúde pública direcionadas a riscos individuais e ambientais identificáveis nesses locais comuns seriam mais benéficas na redução de lesões oculares nessa população.

## INTRODUCTION

Ophthalmic emergencies account for 2.5 million emer gency department visits in the United States yearly^(1,2)^. A recent study determined that ocular trauma accounted for 77.9% of all eye-related emergency room visits, of which 1.4% were admitted primarily for ocular trauma and 6.5% for other injuries^(3)^. Most eye-related hospital admissions result from trauma, 90% of which are believed to be preventable^(4)^. Ocular trauma is a major cause of unilateral blindness in the United States, affecting between 40,000 and 60,000 individuals per year^(1,2)^.

A review of research funded by the Centers for Disease Control and Prevention by Prevent Blindness America estimated that the economic burden of visual impairment in the United States in 2013 was $139 billion (or $6,680 per person per year)^(5)^, which included the costs of acute and long-term healthcare, government-assisted programs, low-vision aids, and loss of productivity for the patient and the designated caregiver. One study estimated a 62% increase in the cost of hospital treatment of ocular trauma from 2002 to 2011 (Abstract PO118 presented at the American Academy of Ophthalmology Annual Meeting, 2015). However, the associated decline in quality of life is harder to quantify.

Several epidemiologic studies of patterns of ocular trauma have been conducted worldwide detailing characteristics specific to the studied community^(1,6-10)^. However, to our knowledge, no study has yet addressed ocular trauma in elderly individuals residing in the Bronx. The Jacobi Medical Center is one of three level I trauma centers in the Bronx and serves around 1.45 million residents, 34.1% of whom are foreign born^^11^^. Of these, 45.4% are Caucasian and 43.4% are African American. Hispanic ethnicity predominates, accounting for 55.1%, compared with 17.6% for the entire United States^(12)^. The current poverty rate in the Bronx is 31.3%^(11)^.

Because population trends have a vast and wide-ranging impact on economic patterns and healthcare delivery decisions and policies, understanding the unique characteristics of certain communities enables appropriate interventions and resource allocation. The older population is experiencing unprecedented growth in the United States. The population aged ≥65 years increased from 3 million in 1900 to 43.1 million in 2012. The US Census Bureau has projected that by 2050, this group will reach 83.7 million, almost doubling the 2012 estimate and increasing from 13% to more than 20% of the population. Chiefly responsible for this growth are the baby boomers, who started turning 65 in the year 2011^(13)^. Boomers surviving to 2050 will be older than 85 years, expanding this segment from 1.9% to 4.5% of the general population^(13)^. Furthermore, the population will become more racially and ethnically diverse, with females continuing to outnumber males. Likewise, the Bronx population is expected to mirror these trends.

Given the preventable nature of ocular trauma, further studies of the epidemiology in the elderly could have significant public health benefits for this susceptible and ever-increasing portion of the population and for the community at large. The purpose of this study was to evaluate the demographic composition of patients and the types, mechanisms, and locations of ocular injury in major trauma admissions among elderly patients presenting to an urban level I trauma center. Analysis of the characteristics of ocular trauma in this subpopulation could assist in appropriate resource allocation and the development of targeted preventative measures to reduce visual disability, dependency, and healthcare costs.

## METHODS

The medical records of patients aged ≥65 years, treated for ocular injuries from January 2008 to June 2015, in the setting of major trauma cases were retrieved from the electronic medical record system of Jacobi Medical Center and retrospectively reviewed. This study protocol was approved by the Institutional Review Board of the Albert Einstein College of Medicine and conducted in accordance with the tenets of the Helsinki Declaration (seventh revision, 2013). Patient consent was waived because the identification of personal details was not part of this investigation. The International Classification of Disease ninth edition-Clinical Modification (ICD-9-CM) codes were used to identify all manifestations of ocular trauma from major trauma admissions as submitted by the Emergency Department to the National Trauma Data Bank (NTDB). These included all the trauma subsets of codes 870, 871, 918, 921, 930, 940, 364, 802, 376, 950, and 951. ICD-CM-E codes were used to determine the mechanism of injury.

Patient demographic data, including sex, age, race, and ethnicity, were obtained from the medical records. For each patient, injury type, mechanism, and location, associated features, intention, injury severity score (ISS), and Glasgow coma score (GCS) were detailed. ISS, a numerical assignment from 1 to 75 to designate the degree of injuries to a patient, was documented according to NTDB categories as follows: 1-8, minor; 9-15, moderate; 16-24, severe; > 24, very severe^(14)^. GCS was also categorized according to NTDB subgroups as mild (13-15), moderate (9-12), or severe (≤8). All patients with complete data, admitted from January 2008 to June 2015 (7.5 years), for major trauma associated with eye injuries were included for analysis, whereas those with incomplete data, duplicate submissions, or not having ocular injuries were excluded.

### Statistical analysis

Once extracted from the electronic notes, the data was de-identified and descriptive analysis was conducted. Data was transformed to binary notation for statistical analysis. De-identified tabulated Excel data (Microsoft Corp., Redmond, WA) was used for descriptive statisti cal analysis. Continuous variables were presented as mean ± standard deviation (SD), median, and interquartile range (IQR), and categorical variables were grouped, and proportions expressed as percentages. Associations between variables were identified using the chi-squared test and the Student’s paired *t*-test. Simple linear and logistic regression analyses were performed to establish the strengths of the associations. All statistical analyses were performed using STATA/MP-12 software (StataCorp LP, College Station, TX), and graphs and tables were generated with Excel software. A probability (p) value of <0.05 was considered to be statistically significant.

## RESULTS

From a total of 13,825 patients admitted for major trauma, 861 (6.2%) had ocular injuries, which included 221 (25.7%) aged ≥65 years. The mean (± SD) age of the elderly subgroup was 80.3 ± 9 years with a median of 79.2 years (IQR=63.8-94.6). Of the 221 elderly patients, 40.7% were males and 59.3% were females. The males were younger than the females (mean age=77.3 [95% CI=75.5-79.1]) vs. 82.4 [95% CI=80.8-83.9] years, respectively, p<0.001; Table 1). With respect to race, 30.8% of the patients were white, 13.6% were black, and 54.3% were identified as “other.” Of those classified as “other,” 67.5% (36.7% of the overall total) were identified as Hispanic (Table 1). Females outnumbered males in all the racial and ethnic categories.

**Table 1:** Basic demographics of elderly patients admitted with ocular trauma (2008-2015)

characteristic	Female	Male	Total
Total ocular injuries	131 (59.3%)	90 (40.7%)	221 (100%)
Race			
White	41	27	68 (30.8%)
Black	21	9	30(13.6%)
Asian	1	1	2 (0.9%)
American Indian	1	0	1 (0.5%)
Other	67	53	120 (54.3%)
Ethnicity			
Non-Hispanic	74	58	132 (59.7%)
Hispanic	53	30	83 (37.6%)
Unknown	4	2	6 (2.7%)
Age (mean, Cl)	82.4 (80.9-83.9)^*^	77.3 (75.5-79.1)^*^	80.3

* *p*<0.0001 (Student’s *t*-test).

The mean ISS was 9.76 ± 9.15 (moderate severity), and the mean GCS was 14.3 ± 2.05 (mild traumatic brain injury). The majority (90.95%) of trauma incidences were unintentional, followed by 4.98% due to assault and 4.07% due to other causes (undetermined/ unknown, medical misadventure, or self-inflicted). The most frequent types of injuries were contusion of the eye and adnexa (68.2%), orbital wall fractures (22.2%), and an open wound of the ocular adnexa (18.1%) (Table 2). Open-globe injuries accounted for only 6.3% of all ocular injuries. None of the injuries were iritis, hyphema, foreign bodies (external cornea), burns to the eye/adnexa, or cranial nerve injury.

**Table 2:** Type of injury in elderly patients admitted with ocular tTrauma by sex (2008-2015)

		Female	Male	95% CI	p	
Type of Injury Contusion of the eye and adnexa		92 (70.8%)	58 (64.4%)	0.422-1.329	0.323	
Open wound of the ocular adnexa		27 (20.6%)	13 (14.4%)	0.315-1.342	0.244	
	Orbital fracture	20 (15.3%)	29 (32.2%)	1.378-5.053	0.003	
	Superficial injury	18 (13.7%)	18 (20.0%)	0.766-3.215	0.218	
	Orbital hemorrhage or edema	18 (13.7%)	13 (14.4%)	0.491-2.289	0.882	
	Open wound of the eyeball	7 (5.3%)	7 (7.8%)	0.505-4.416	0.468	
	Subconjunctival hemorrhage	5 (3.8%)	6 (6.7%)	0.532-6.088	0.344	
Other		2 (1.5%)	2 (2.2%)	0.203-10.602	0.705	
Injury to the optic nerve and pathways		0 (0%)	1 (1.1%)	-	-	
Lens subluxation		0 (0%)	1 (1.1%)	-	-	

Table of simple logistic regression analysis. Males had greater odds of orbital fractures than females, and all the other injuries were not significantly different in frequency between the sexes.

Males had a 2.64-fold greater incidence of orbital wall fractures than females (95% CI=1.38-5.05, p=0.003). Of the other injuries, there were no significant differences in frequencies between males and females. No other relationships were noted with respect to race, sex, or ethnicity with a specific injury. However, those with orbital wall fractures had a higher mean ISS than those without (17.5 [95% CI=14.1-20.9] vs. 7.7 [95% CI=6.88.6], respectively, p<0.0001).

**Figure 1:**
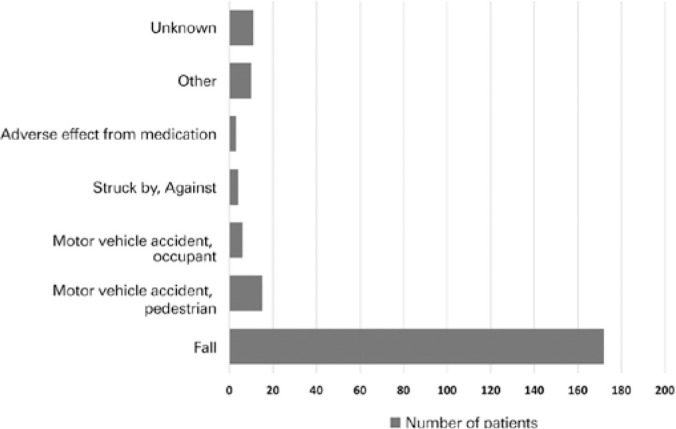
Mechanisms of injury in elderly patients admitted with ocular trauma (2008-2015). Falls far outnumbered other mechanisms of trauma in the elderly.

**Figure 2:**
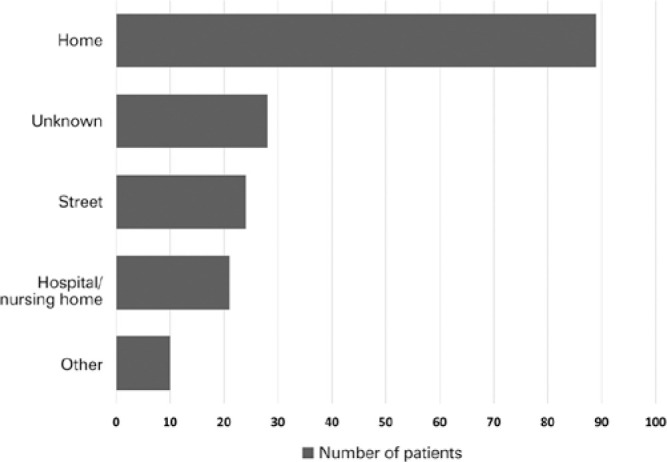
Location of injury in elderly patients admitted with ocular trauma (2008-2015). Homes and nursing home settings were the most common locations of trauma.

The most common mechanisms of injury were falls (77.8%) and pedestrian/motor vehicle accidents (6.8%) (Figure 1). Females had a 1.91-fold greater risk of eye trauma from a fall than males (95% CI=1.01-3.62, p=0.048). Documented locations of falls were at home (51.7%), on the street (13.9%), and in hospitals/nursing homes (12.2%) (Figure 2). Those falling at home were older than those falling at other locations (mean age=83.4 [95% CI=81.8-85.4] vs. 78.8 [95% CI=77.080.6] years, respectively, p=0.0002). Those falling in the bathroom were also older than those not specified as falling in the bathroom (mean age 91.25 [95% CI=85.397.2] vs. 80.7 [95% CI=79.4-82.0] years, respectively, p=0.0001; Table 3). In this elderly population, 14.5% were diagnosed with visual impairment or blindness, as defined by criteria of the World Health Organization^(15)^, and 33.1% were diagnosed with cognitive impairment (dementia or psychiatric disorders). The clear majority of falls occurred at ground level (syncope, loss of balance, tripping, and stumbling), although stairs were implicated in 15.7% of cases.

**Table 3:** Significant differences in fall-related ocular trauma in elderly patients (2008-2015)

Characteristic		Difference	Odds ratio	95% CI	p	
Sex Female		Females had greater odds of eye injury from falls	1.91	1.01-3.62	0.048	
Male		Males had greater odds of orbital fractures	2.64	1.38-5.05	0.003	
	Age		Mean (years)			
	Home	Those falling at home were older than those falling elsewhere	83.4	81.8-85.4	0.0002	
	Not home		78.8	77.0-80.6		
	Bathroom	Those falling in the bathroom were older than those falling elsewhere	91.25	85.3-97.2	0.0001	
	Not Bathroom		80.7	79.4-82.0		
	Alcohol	Falls associated with alcohol intoxication occurred in younger patients	72.15	68.9-75.4	0.0002	
	No alcohol		82	80.7-83.3		
ISS			Mean			
Orbital fracture		Patients with orbital fractures due to falls had a higher ISS	17.5	14.1-20.9	<0.0001	
No orbital fracture			7.7	6.8-8.6		

Simple logistic regression of variances in falls: falls are the most common mechanism in females, who had greater odds of falls than males. Males had greater odds of orbital injuries, which were most commonly associated with severe ISS. Home and bathroom falls occurred in older patients, whereas alcohol-related falls occurred in younger patients. CI= confidence interval; ISS= injury severity score.

Alcohol consumption was associated with 6.43% of falls, and those whose falls involved alcohol were younger than those with no mention of alcohol (mean age=72.15 [95% CI=68.9-75.4] vs. 82 [95% CI=80.783.3] years, respectively, p=0.0002). Furthermore, males were more likely to fall while intoxicated than females (p<0.0001). Fall-related orbital fractures had a higher mean ISS than orbital fractures from other mechanisms (17.5 [95% CI=14.1-20.9] vs. 7.7 [95% CI=6.8-8.6], respectively, p<0.0001). Falls associated with stairs and alcohol consumption were not associated with higher ISS (p=0.29 and 0.72, respectively). A summary of all the statistically significant variables associated with fall-related ocular trauma is presented in table 3.

## DISCUSSION

This hospital-based study showed that the number of elderly patients admitted with major trauma associated with ocular injuries (25.7%) exceeded the proportion of elderly people in the Bronx (14.9%)^(11,12)^. As with the US general population, most patients were female. The most common injuries were superficial adnexal contusions and orbital wall fractures. The latter occurred mostly in males and was associated with a higher ISS, within the NTDB designation of moderate to severe injury^(14)^. Open-globe wounds were less common, occurring in only 6.3% of cases. Most injuries occurred in the Hispanic population, reflecting the demographic makeup of the Bronx^(10)^. The clear majority of injuries were caused by falls, with almost 64% occurring at home or in a nursing facility. This group was found to be older than those falling outside the home environment, a finding that might reflect that older, more infirm patients tend to be confined to home/nursing facilities. In all locations, ground-level falls were the most common type, although stairs were implicated in a minority of patients. Other associated factors were poor vision, cognitive impairment, alcohol intoxication, and syncope.

Ocular trauma in the elderly has its own unique challenges and is associated with a higher risk of globe rupture and poorer visual outcomes when compared with other age groups^(16-21)^. Few studies have investigated the nature of ocular trauma with a focus solely on elderly patients, but most have found that falling is the most frequent mechanism of injury^(10,16-21)^. Falls are a leading cause of traumatic morbidity and mortality in the elderly due to risk factors that include poor vision, previous falls, fear of falling, frailty, weakness, poor mobility, imbalance, cognitive deficits, living alone, environmental hazards, and multiple medical comorbidities^(22-24)^. Visual disability resulting from fall-related trauma will likely contribute to subsequent falls and a cycle of emergency room recidivism.

Ocular trauma in the elderly is associated with a poor prognosis and a higher rate of hospitalization than trauma in younger age groups^(17-20)^. In a study by Andreoli et al., 4% of open-globe injuries were due to falls in the nongeriatric group compared with 65% in the geriatric group^(19)^, implying an increased incidence of rupture from falls with advancing age. They attributed this higher incidence in the geriatric population to the likelihood of previous eye surgery that weakened and rendered the eyes more susceptible to rupture. Most studies have also identified falls as the leading mechanism of injury in this age group^(15-19,23)^. Falls are the leading cause of all trauma admissions in the United States. A review of the NTDB reports from 2008 to 2015 indicated a linear increase in the incidence of falls from 32.3% to 43.4%, with falls becoming the number one reason for trauma admissions and death, ahead of motor vehicle accidents and firearms^(14)^. A recent NTDB report documented that falls accounted for 78.8% of all injuries in the elderly. The fall rate of 77.8% in the present study agrees with the NTDB rate but is higher than the fall rate among the elderly reported in previous ocular trauma studies^(10,17-20)^. This consistency with the NTDB rate might reflect similarities in the study populations of hospitalized patients. Other studies might have included both inpatient and outpatient trauma cases with a different spectrum of trauma mechanisms. A recent study that lends support to this contention is the Helsinki Ocular Trauma Study. Sahrarav and his team^(21)^ surveyed elderly (>60 years) patients with ocular trauma. Although they confirmed that falls were the most frequent and serious mechanism of injury, the overall rate of falls was only 22%. However, when they analyzed the 14% of patients who were admitted, they found that 69% had sustained an injury from a fall, a figure that compares favorably with our findings^(21)^. Likewise, they found that the home or institutions were the most frequent locations of injury.

Although beyond the scope of this report, a discussion about interventional methods to address the high number of ocular injuries in the elderly would likely have the greatest impact. A number of reports have documented effective fall-reduction strategies that include individual risk assessments and the institution of targeted interventions such as various exercise regimens and identifying and reducing environmental hazards^(22-24,26-28)^. Although our findings agree with previous ocular trauma studies and the latest NTDB report, the development of preventative interventions would likely have to be tailored to the specific challenges of the unique demographic characteristics of the targeted population. Our study identified a large proportion of Hispanic patients and a higher likelihood of injuries occurring in the home/nursing home environment than at other locations. Thus, targeted interventions aimed at reducing individual patient-specific risk factors and modification of identified home-related environmental hazards while incorporating linguistic and cultural considerations would likely be the most effective strategy to reduce the risks of falls and ocular injuries in this population.

The limitations of this study include the inherent restraints of a retrospective design reliant on database records to document injuries. The extracted data were only as accurate as the assessment and documentation of the submitting physicians. Possible ICD-9-CM misclassifications might also have negatively impacted the veracity of our findings. We noted that the cataloging of risk factors differed from patient to patient. An important example was the lack of uniform documentation of visual acuities at baseline and presentation, a vital risk factor for falls in the elderly. Visual functions, including Snellen visual acuity, visual fields, contrast sensitivity, and stereopsis, are important for visualizing and avoiding environmental hazards and for providing cues that help stabilize balance, thus influencing the incidence of falls^(26-28)^. A prospective population-based observational study design using a standardized assessment protocol developed by a multidisciplinary team that catalogs all contributing risk factors, clinical evaluations, and submission of data would help to address the study limitations and provide stronger evidence allowing for comparisons with other studies.

In conclusion, our study characterized patterns of ocular trauma in the elderly within the urban setting. We clearly illustrated that in the urban setting, ocular trauma occurred at a rate higher than the proportion of elderly residents within this population, and falls were by far the most common mechanism. Most falls occurred in the home/nursing home environment. Our findings are an important step forward in understanding ocular trauma in the elderly population, while providing groundwork for the development of public health strategies to prevent injuries that are associated with higher morbidity and mortality in this vulnerable and expanding age group.
